# Semiochemicals Associated with the Western Flower Thrips Attraction: A Systematic Literature Review and Meta-Analysis

**DOI:** 10.3390/insects14030269

**Published:** 2023-03-08

**Authors:** Marco A. Díaz, Coralia Osorio, Ericsson Coy-Barrera, Daniel Rodríguez

**Affiliations:** 1 Biological Control Laboratory, Facultad de Ciencias Básicas y Aplicadas, Universidad Militar Nueva Granada, Cajicá 250247, Colombia; 2 Departamento de Química, Universidad Nacional de Colombia, Bogotá 14490, Colombia; 3 Bioorganic Chemistry Laboratory, Facultad de Ciencias Básicas y Aplicadas, Universidad Militar Nueva Granada, Cajicá 250247, Colombia

**Keywords:** *Frankliniella occidentalis*, pheromones, kairomones, lures, sticky traps, monitoring, pyridine-related compounds

## Abstract

**Simple Summary:**

Over the past two decades, researchers around the world have conducted studies to identify volatile compounds that serve as attractants for one of the most important agricultural pests, the western flower thrips (WFT). In this review, the preferred reporting items for systematic reviews and meta-analyses (PRISMA) guidelines were used to find published studies and to extract the information generated worldwide to determine the overall attraction of the reported compounds using a meta-analysis approach. This systematic review shows that the most-used compounds worldwide are those based on pyridines, which show a high attraction ratio. Methyl isonicotinate is the most studied of these compounds. Data from the meta-analysis accurately estimated the potential attraction of other novel compounds that could be used for integrated pest management strategies, such as monitoring WFT populations or mass trapping. A call for new research evaluating and developing new products with these novel compounds is made.

**Abstract:**

The study of the semiochemicals of the western flower thrips (WFT), *Frankliniella occidentalis*, Pergande (Thysanoptera: Thripidae), is a relevant topic that spans the last two decades. Approximately a hundred articles published on this subject from 2000 to 2022 can be found in academic databases, representing approximately 5% of the research on this important pest. These topics have generated a platform for novel research with a high potential for development. However, to move on to a new research step, an effectiveness evaluation of the compounds discovered so far is necessary. This review conducted a systematic analysis of the research focused on the semiochemicals (kairomones, pheromones, and attractants) for this pest. Papers from the past three decades on WFT attraction to semiochemicals were collected from databases using the Preferred Reporting Items for Systematic Reviews and Meta-Analysis (PRISMA) guidelines. The number of individuals attracted to compounds was extracted from the papers and compiled for analysis. With this information, an attraction ratio was calculated. Forty-one possible attractants were found in the literature, with methyl isonicotinate being the most-studied compound so far, with the third-highest attraction ratio. *δ*-Decalactone was the compound with the highest attraction ratio, but it was one of the least studied. A meta-analysis of the WFT choosing proportion was performed for the compounds with more trials found in the literature. The predicted mean choice percentages for methyl isonicotinate (MIN) and Lurem-TR, the MIN’s commercial product, were 76.6% and 66.6%, respectively. There was a convergence among the analyzed studies showing a high degree of research focus on the same group of nitrogen-containing compounds (mainly the pyridine structure). These findings call for future research to diversify the discovery and evaluation of attractive compounds in this relevant study area.

## 1. Introduction

The western flower thrips (WFT), *Frankliniella occidentalis* Pergande (Thysanoptera: Thripidae), has been recognized as a pest of agricultural importance since the 1970s in America [1,2,3]. It has obtained the status of plague worldwide [4] due to the fact of its wide range of hosts. It has been reported that WFT attacks more than 600 species of plants, including ornamental and horticultural crops, in both the greenhouse and the open field [3,5]. The relevance lies in the damage it can cause, reducing the production or quality of products [6,7,8], inducing senescence of inflorescences [9], and being a tospovirus supervector, especially the *tomato spotted wilt virus* (*TSWV*) [10,11,12,13]. Records on the total damage caused by thrips are difficult to obtain, as growers are reluctant to inform on the presence of this pest and the cost associated with controlling it, and because it is challenging to quantify the cost and losses with a unique method [14]. For green beans, it was determined that the losses are between 10.74% and 36.65% [15].

Since the early 2000s, the costs of controlling this pest have increased over time due to the generation of populations that are resistant to the market’s most widely used commercial synthetic insecticides [16]. Broad-spectrum and narrow-spectrum insecticides have been used for WFT management, but the frequent application of these insecticides without a rotation scheme or based on economic damage thresholds has led to the development of WFT resistance to the active ingredients of most chemical classes [17]. Resistance mechanisms are an essential study topic, mostly related to incorrect pesticide doses, indiscriminate mixtures of several pesticide types, or limited necessary rotations of chemical pesticides [5].

These drawbacks have motivated the search for alternative solutions to be used simultaneously with the standard control methods to regulate WFT natural populations in economic crops. One of these alternatives is behavioral control [17], which involves the manipulation of adult insect behavior using traps amended with synthetic semiochemicals commonly related to naturally occurring compounds produced, emitted, and perceived by animals and plants for chemically mediated communication [18].

Some authors classified these compounds in terms of the responses they elicit from insects (attractants, repellents, arrestants, deterrents, or stimulants) [19,20]. Moreover, considering the communication level, semiochemicals are divided into allelochemicals and pheromones. Allelochemicals are essential for interspecific communication, while pheromones are used for intraspecific communication [21]. It has been demonstrated that plants produce allelochemicals to defend against attacks from their pests because emitting volatile organic compounds (VOCs) allows them to repel pests or attract natural enemies before or during the attack [22]. In addition, pest insects recognize part of the volatiles, constitutively or inductively produced by plants, for their benefit. Such compounds are called kairomones.

Despite its potential, a preliminary search for literature on the Web of Science (WoS) before 2013 revealed that the research percentage directed toward developing WFT-related semiochemical-based strategies was 1.1% of the total number of thrip management-related studies [23], showing an apparent low interest in this field. In contrast, by 2022, this percentage increased to 4.8% of the total research volume in the literature on the semiochemicals associated with WFT (WoS on October 2022, *n* = 2104 articles). In addition to the low volume of research, it mainly converged in the study of a particular group of 4-pyridyl carbonyl compounds reported as attractants [23,24,25,26,27,28,29] and the same sticky-trap types, usually square and blue [18,30,31,32].

In this context, the implementation of integrated pest-management strategies based on semiochemicals has developed slowly and has been focused mainly on monitoring insect pests, despite the great potential and uses of this technology found in the last 25 years of research [33]. Farmers tend to be disinclined to implement traps amended with attractants [34] due to the lack of accurate information on capture performance, or the information supplied by distributors is sometimes ambiguous or misunderstood by trap users. These facts may be partially caused by the different attraction levels reported for the same compound when evaluated in the laboratory [35], the open field [28], or at different times in the same trials [23] as criteria for utilization under crop conditions.

To achieve a better understanding of semiochemicals attraction in the laboratory or increased capture for traps in the field, this review aimed to conduct a PRISMA-guided systematic literature review, from 2000 to 2022, on the chemicals associated with WFT attraction, performing a meta-analysis to determine the more accurate attractiveness by using these compounds. For the purpose of this review, the term WFT will be taken to refer to the pest complex (biotypes and subspecies recognized as *F. occidentalis*). In addition, the term “attraction” will be used for both attractants and arrestants.

## 2. Methods

### 2.1. Systematic Review

This systematic review used the procedure established in the PRISMA guidelines [36]. The first step was screening the literature from 2000 to 2022 in the Web of Science (WoS), Scopus, Science Direct, ProQuest, and Springer databases to identify possible articles. The commands that were used varied according to the language of each database, maintaining the same structure. For instance, the command used in WoS was TS = (Frankliniella occidentalis OR Western Flower Thrips) AND TS = (semiochemical * OR pheromone * OR kairomone * OR lure). For Scopus or other databases, the commands *TITLE-ABS-KEY(semiochemicals OR trap OR lure) AND TITLE-ABS-KEY(*“*Frankliniella occidentalis*” *OR* “*Western Flower Thrips*”*)* were employed.

Duplicates between the databases were recognized and eliminated from the entire set of retrieved articles using logical functions in MS Excel^®^ (Microsoft Corp., Redmond, WA, USA) (IF, and XLOOKUP). Subsequently, screening was carried out using two inclusion criteria: (1) type of paper—research articles, review articles, and short communications; (2) inclusion of a binomial test—evaluation of natural or synthetic compounds on the behavioral response of WFT, especially binomial-type tests that evaluate the compound attractiveness compared to a control (e.g., without the compound). Articles submitted in languages other than English (e.g., Spanish and Portuguese) were excluded, as well as scientific notes, web pages, technical reports, and brief/long reports in abstract books of scientific conferences. Only peer-reviewed articles were included, so gray literature and books were excluded. The reference list of the identified articles was reviewed to avoid a possible loss of relevant articles.

### 2.2. Data Extraction

From the set of articles retrieved from the databases, a matrix was built with the following data: publication DOI, name of the compound, assay type, environment condition (laboratory or field), semiochemical type (kairomone lure, pheromone, or repellent), and sex and age of evaluated individuals. Mainly, two experiment types were found in the literature, i.e., olfactometry under laboratory conditions or sticky traps in the field. All selected papers measured the compound’s attractiveness by performing a binomial experimental design involving two treatments, i.e., one with the compound and a control treatment without the compound. The response variable in this kind of research was the number of insects captured by a trap in each treatment or the number of individuals choosing each treatment in the olfactometry experiment. The information found in each paper regarding the number of individuals captured in the compound-containing treatment and the number of total individuals evaluated in the experiments was compiled in the matrix used for the meta-analysis.

The extraction method for the numerical data was carried out as follows. First, the data were extracted directly by searching in the article text or included tables. Subsequently, the total number of individuals evaluated in each experiment was registered in the matrix if any of the following two conditions were satisfied: (1) the number of individuals choosing each treatment or (2) the proportion of individuals choosing the treatment with the test compound. If the authors did not report the total number of test individuals, those individuals attracted in the two treatments (i.e., with and without the test compound) were summed up to obtain the total.

The extraction method for the graphical data was performed as follows. Usually, the articles’ data were registered as bar or scatter plots involving data dispersion, such as confidence intervals or standard deviation. To extract this data, we used the online software WebPlotDigitizer [37]. The value of the points or bars drawn on the graph was calculated, considering the scale on the x- and y-axis reported by the authors. When the value in the plot was a proportion, it was transformed into the number of individuals using the authors’ reported total (*n*) of individuals.

### 2.3. Meta-Analysis

The extracted data allowed us to calculate the attraction ratio calculation, defined as the number of insects attracted by the test compound–containing treatment divided by the number of individuals attracted by the control treatment. This ratio was interpreted as the *n* times (*n*x) that the test compound attracted more individuals than the control. This parameter is mainly descriptive and is typically reported by companies that commercialize semiochemicals as an effectiveness measure. Therefore, the present study used it for descriptive analysis. Second, from the same data, another parameter was calculated using the number of individuals choosing the test compound and divided by the total of individuals in the bioassay or trapped in the field. These data were defined as the proportion ratio and were the variables used for the meta-analysis. There is a mathematical relationship between these two parameters, as expressed in Equation (1):

(1) $$AR=\frac{P}{1-P}\;\;\;\;\;\;\;\;\;$$

where *AR* is the attraction ratio and *P* is the proportion of individuals choosing the test compound. Note that the value of *Ar* tends to infinity when a higher proportion value has occurred.

In several articles, more than one trial was performed with the test compound. Therefore, each trial was taken as an observation to perform the meta-analysis. Only three compounds were used to perform the meta-analysis because they were the only ones with enough trials to perform the statistical tests. These compounds were methyl isonicotinate (MIN) (*n* = 27), MIN’s commercial Lurem TR (*n* = 28), and *p*-anisaldehyde (*n* = 10). The meta-analysis was performed with the R package “metafor” [38] in a random effects model using the proportion of individuals choosing the test compound as the response variable. To avoid zero-biased data, the double arcsine transformation of the proportion was used. Forest and funnel plots were constructed to analyze the results, and an influence test was performed to detect studies that introduced extra residual heterogeneity into the model. Finally, a meta-regression analysis was performed, using the assay type (olfactometer vs. sticky traps) and the assay conditions (laboratory vs. field) as moderators.

## 3. Results

### 3.1. Findings about Western Flower Thrips General Literature

**Figure 1:**
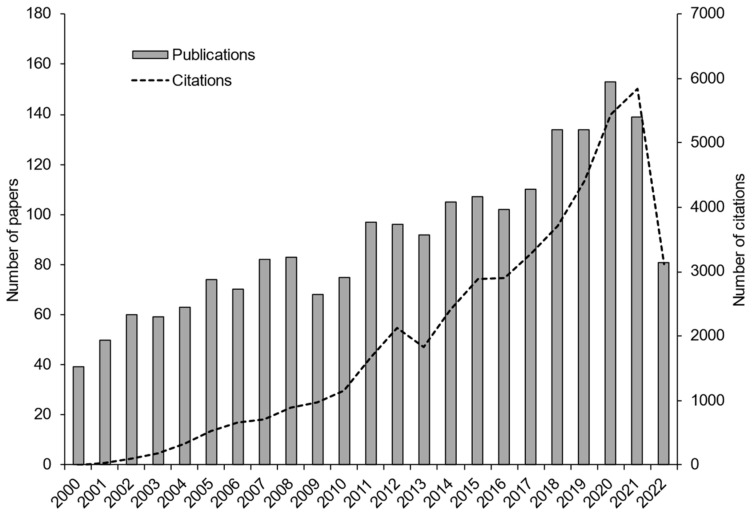
Record of publications and citations of articles related to western flower thrips found in the Web of Science database from 2000 to 2022.

Since the last decade, studies related to WFT have been increasing. A simple search in the Web of Science database using the command TS = (*Frankliniella occidentalis* OR western flower thrips) retrieved 2104 results, covering the period from 2020 to 2022, with an *h*-index of 84 and a growing trend in the citation number of these articles (Figure 1).

**Figure 2:**
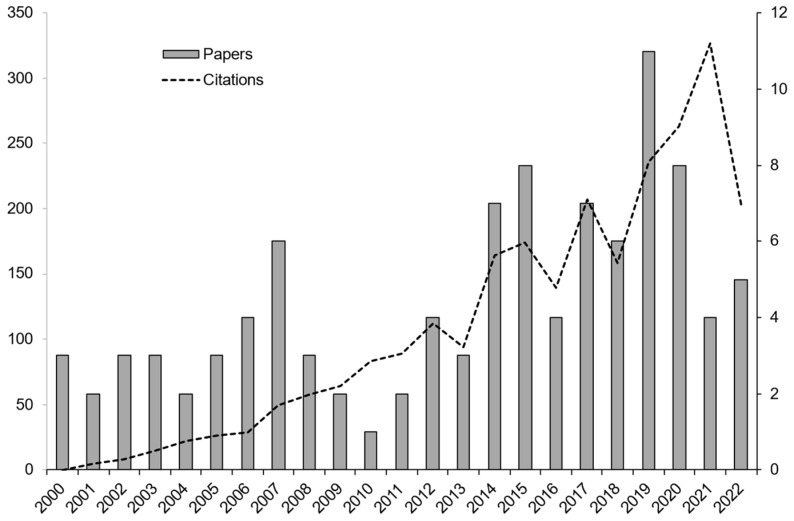
Record of publications and citations of articles related to the WFT complex and semiochemicals found in the Web of Science database from 2000 to 2022.

The systematic search in the Web of Science database produced 101 publications referring to WFT semiochemicals, considering only research or review articles from 2000 to 2022. During this period, while screening the papers’ titles and abstracts found in the literature, two relevant stages could be identified in the WFT-related publications and semiochemical studies: one from 2000 to 2009 and another from 2010 to 2022 (Figure 2). The first period was focused on the search and evaluation of compounds (mainly pyridine-related compounds). Hence, publications that were related mainly to implementing and evaluating the identified compounds during the first period could be observed in the second period—mostly MIN and related commercial products.

### 3.2. Literature Related to Semiochemicals Associated with WFT

**Figure 3:**
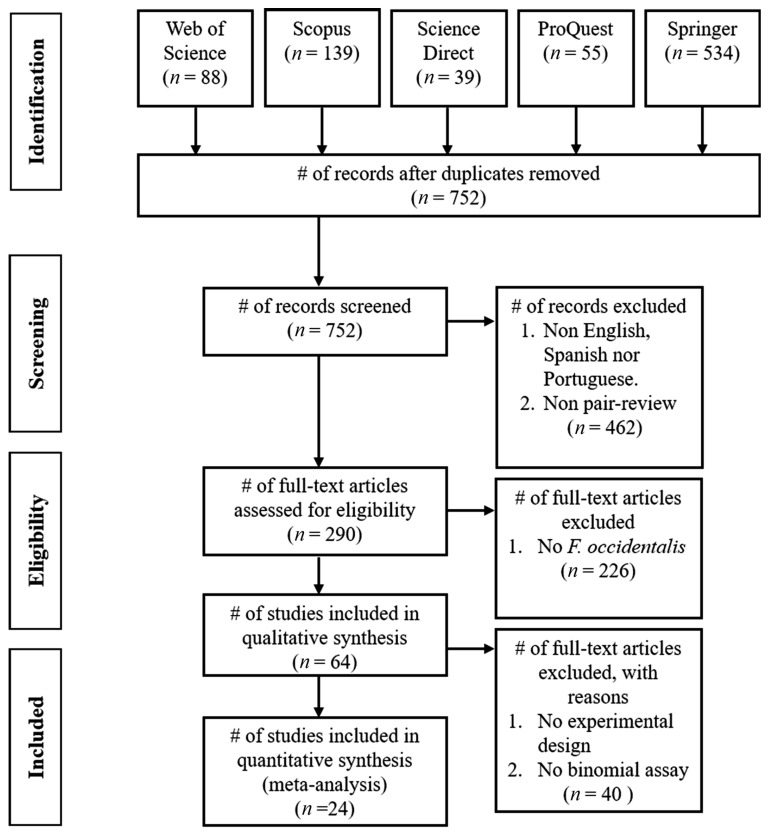
PRISMA workflow for collecting papers in the databases. Numbers in parenthesis are the paper number included or excluded for each workflow step. # = number.

A total of 855 records was found in the consulted databases. Within this group, 103 articles were duplicates, so the final set, after identification, included 752 articles. Figure 3 describes the process of searching and selecting items using the PRISMA flow chart with the criteria of exclusion at each stage.

#### 3.2.1. Kairomones, Attractants, or Lures

Although in the context of integrated pest management the term kairomone usually referred to any compound used as an attractant for pests, this concept does not meet the ecological criteria implied in the kairomone definition (i.e., a semiochemical that harms the emitter in one species and benefits the receiver in a different species [33]), since the emitter is not a plant or other species but a trap or dispenser. Some authors have avoided the term kairomone and used the terms non-pheromone semiochemicals or lure when referring to field evaluation [39]. However, the interpretation of kairomones can become complex for persons who are unfamiliar with scientific terminologies, such as farmers or producers. The term *attractant* is defined as a “chemical causing a responder to make movements oriented towards the stimulus source” [19,20]. Although this term does not cover all possible behavioral responses of flying thrips, it is used in this study, for practical understanding.

One of the first studies evaluating the compound attractiveness to WFT was based on the implementation of field traps impregnated with a lure previously recognized in other thrips species, i.e., *Thrips obscuratus* (Crawford) in New Zealand. Such a compound was ethyl nicotinate (EN), previously found in peach fruits during the ripening process, a plant in which thrips have constantly been found [40]. Using chromatic traps, it was determined that this compound had the potential to attract 20.52 times more individuals than the control traps. However, these preliminary studies lacked the standard criteria for evaluating this compound type in the field, such as trap disposition, loading amount, and dispenser release rate.

Later, at the end of the 1990s, the “Y-tube” olfactometer was developed to analyze the thrips behavior response under controlled conditions [41]. With this bioassay, it was possible to determine the attraction level of plant VOCs and compare the behavioral responses of different compound types, such as benzenoids, monoterpene isoprenoids, sesquiterpene isoprenoids, phenylpropanoids, nicotinoids, and the mix of such compounds that is present in essential oils [42].

Among the retrieved 64 publications from the database concerning WFT and semiochemicals, only those that conducted binomial-type evaluations were selected, either under laboratory conditions or field conditions. The most-used experiments were chromatic traps amended with and without compounds and the Y-tube olfactometry bioassay. These studies contained information on the number or percentage of individuals choosing treatment or those captured in the traps. The attraction ratios were then calculated from these data (See Section 2.3).

Table 1 summarizes the entire set of WFT possible attractants investigated in these studies. This information was compiled with the aim of determining their attraction ratios, based on their former categorization as attractants in the original publication. In this context, a reinterpretation or reassessment of the compound function is beyond the scope of this systematic review. These compounds have been used in traps that integrate colors and shapes for field capture or evaluated as attractants for the mobile states of WFT through olfactometry. In this regard, the mean attraction ratio was calculated without differentiating the trials carried out with traps or the olfactory bioassays.

In the research carried out to date, a total of 39 compounds have been evaluated as WFT attractants (Table 1). Among these compounds, the most representative chemical class corresponded to monoterpenoids (MT), with eleven compounds evaluated in various studies, followed by pyridines (8), aromatics (7), and lactones (6). Three sesquiterpenes (ST), two terpene derivatives (TD), a phenylpropanoid (PP), and an allylbenzene (AB) were also evaluated, comprising the chemical classes with minor representatives explored as WFT attractants. Of the total number of evaluated compounds, 31 exhibited an attractive effect when compared with the control treatments, mainly pyridines (Py, 8) and monoterpenoids (8). It can also be inferred that the nitrogen-containing compounds, mainly with a pyridine moiety, were the most-evaluated, either in olfactometry tests or field conditions using traps.

The compound MIN presented the highest number of trials within these compounds, with an attraction ratio of ca. 5*x*. This means that this compound exhibited a five-fold greater attracting capacity than the control (i.e., without the compound) in the evaluated trials. Likewise, the ethyl isonicotinate compound presented a six-fold (6*x*) attraction ratio but involved few evaluation tests. It should be noted that the lurem-TR, the commercial version of pyridimine-based compounds, has an attraction ratio of ca. 2.5*x*, likely because most of the tests were carried out in the field, where other stimuli play a role in the behavioral response of individuals towards traps. With *δ*-decalactone, an attraction ratio of ca. 6.5*x* was calculated, which was the highest among all of the compounds. Still, it is one of the least evaluated, with only three trials reported in the literature.

In the case of geraniol, *γ*-decalactone, and *o*-anisaldehyde, attraction ratios of ca. 5*x*, 5*x*, and 4*x* were obtained from three, three, and one1 trial(s), respectively, suggesting that additional research related to these compounds is needed. On the other hand, the compound with the lowest attraction ratio was *cis*-jasmone, with a value of 0.55*x*, which indicates that, when averaging the trials, this compound did not generate an attractive behavioral response from the evaluated individuals. Other compounds that presented this same phenomenon (attraction ratio less than 1) were sabinene and *trans*-*β*-ocymene; however, this decreased attraction value compared to the control should not be taken as a repellency effect, as the type of binomial test used in these experiments was not designed to evaluate such a behavioral phenomenon.

#### 3.2.2. Pheromones

Regarding the study of WFT pheromones, it has been reported that males produce volatile compounds for the aggregation of both females and males [51,52,53,54,55]. This fact avoids double-energy expenditure in the reproduction and dissemination of the species [56]. The compound identified as the male-produced aggregation pheromone of WFT was neryl (*S*)-2-methylbutanoate [55]*,* which is commercialized under the name Thripher^®^ (Biobest, België) and attracts both female and male adults. However, it has been determined that an aggregation pheromone of WFT is composed of two substances involving a major component (i.e., neryl (*S*)-2-methylbutanoate) and a minor component (i.e., *(R)-*lavandulyl acetate), which can occur with a ratio ranging from 1:0.8 to 1:5 [54,55,57]. These compounds can be found not only in *F. occidentalis* but also in related species such as *F. intonsa* [52], and they are produced and released when males conform to large groups and sexual competition by females takes place. In addition to the aggregation pheromone, compound 7-methyltricosane has been identified as a possible contact pheromone [58]. Recently, researchers found that an unknown pheromone produced by WFT males could be involved in an anti-aphrodisiac behavior for avoiding mating with non-virgin females. [59]

The alarm pheromone identified for WFT is composed of decyl acetate and dodecyl acetate, which are produced by immature stages [51] and are emitted through anal excretion when the larva is attacked [60,61]. When the alarm pheromone is emitted, the other larvae quickly seek refuge in different plant structures to avoid encounters with the natural enemy [62]. The alarm pheromone can also influence the behavior of other adults, preventing them from reaching the plant [63]. Like kairomones, pheromones have been evaluated as part of a strategy for integrating WFT attraction and control in crops [24]. Therefore, some studies integrate chemical control with pheromones [64] or improve biological control with predators [22,65,66]. However, the most frequent use of pheromones is related to trapping for monitoring purposes [23,46,52,67,68].

The tests for evaluating the attraction of the aggregation pheromone were carried out similarly to the tests applied to kairomones, i.e., a comparison between a control (without the pheromone) and a pheromone-containing treatment. This information was used to calculate an attraction ratio between these treatments. In addition, the trials were conducted with living individuals to corroborate their attraction to their conspecific individuals and to evaluate the gender or age influence [69] (Table 2).

The number of studies evaluating pheromones is less than the number of those evaluating other attractants. Several tests in the literature were conducted under laboratory conditions in olfactometry, either with adult males or with the compounds they produce. The commercial product Thripline^®^, manufactured from the mixture of the compounds recognized as pheromones (i.e., neryl (*S*)-2-methyl butanoate and (*R*)-lavandulyl acetate), produces an average attraction ratio of 2.49*x*. In contrast, the evaluation of the individual compounds produced a lower attraction ratio. These facts indicate that pheromone-based products could almost double the number of individuals attracted or trapped. It is unclear if the Thripline^®^ product is solely based on these pheromonal compounds or includes other substances [46].

### 3.3. Meta-Analysis of Methyl Isonicotinate (MIN), Lurem-TR, and P-Anisaldehyde

Among the set of reported semiochemicals, MIN [70] has been the most studied compound [39]; it is currently marketed under the name Lurem-TR^®^ (Koppert, The Netherlands). Its use is recommended at doses of one dispenser per 100 m^2^ for 42 days. Considering that the Lurem-TR product is formulated as a mixture of MIN and some excipients, a meta-analysis was carried out using the data found in the articles containing information on the compound and the commercial product, whose assays for each product were independently performed.

**Figure 4:**
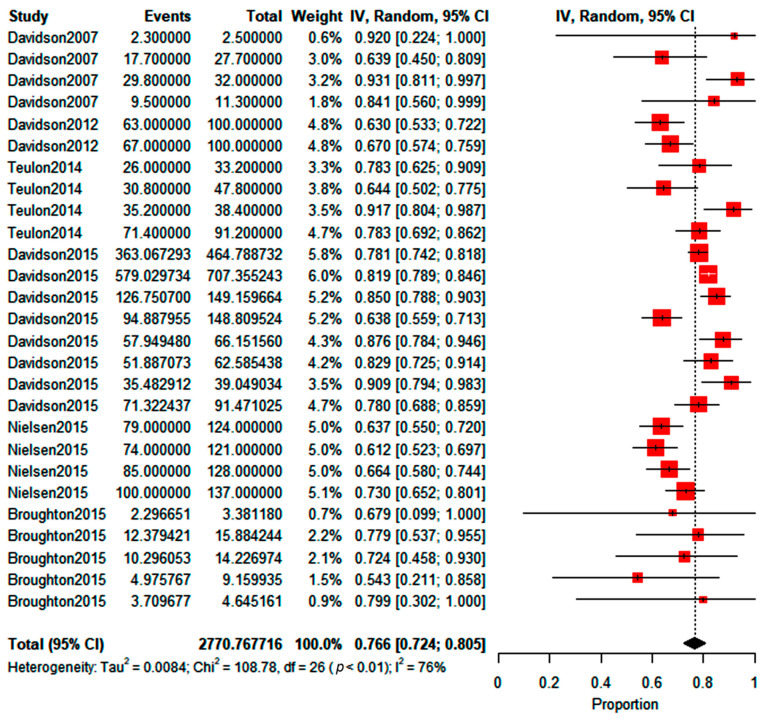
The forest plot for methyl isonicotinate shows the WFT choose proportion (random effects model) between treatment with the compound and the control. Red boxes represent the point estimate.

According to the manufacturer and previous studies [26,28,39], this product can attract up to 14 times more individuals than the control (attraction ratio of 14x). Still, the attraction ratio calculated in this review, using the collected data, reached up to five-fold more than the control, as seen before. Moreover, the meta-analysis with trials recovered from the databases (*n* = 27) showed that the predicted WFT proportion choosing the treatment, compared to the control, was between 0.724 and 0.805. According to the reviewed studies, this outcome is the generalized percentage attraction range of the test compound, with a 76.6% predicted mean choice percentage. This value range for the individual proportion choosing the compound-amended treatment (found in the meta-analysis) translates into an attraction ratio (Equation (1)) between 2.62*x* and 4.13*x*, the latter value being the maximum attraction ratio for these compounds. The forest plot showed that most trials had similar weights, but the heterogeneity between the studies was high (I^2^), indicating a high variability between the studies that were carried out (Figure 4). Moreover, the chi test in the forest plot shows that the overall effect estimate of 76.6% (diamond in Figure 4) was statistically significant.

**Figure 5:**
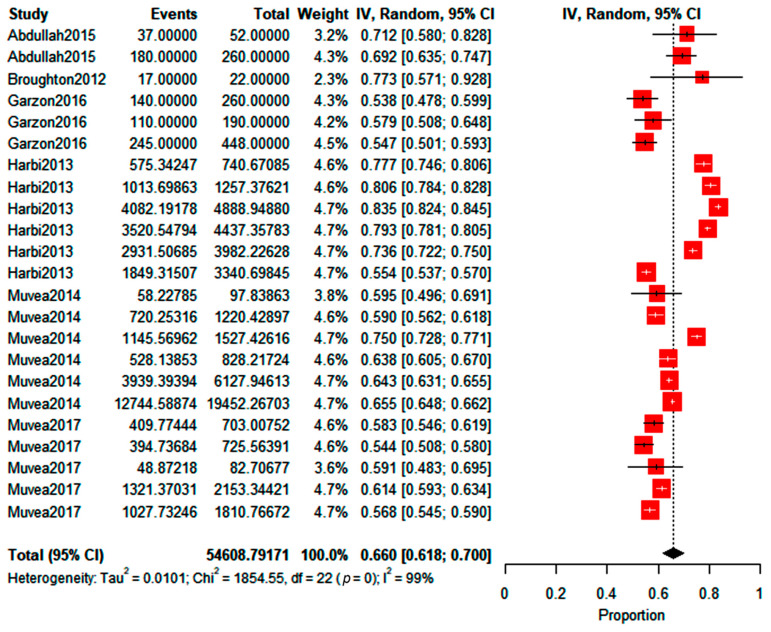
Forest plot for Lurem-TR showing WFT choosing proportion (random effects model) between treatment with the compound and control. Red boxes represent the point estimate.

In the case of trials using Lurem-TR (i.e., MIN with excipients (*n* = 22)), the predicted WFT choosing proportion was found to be between 0.61 and 0.7. According to the reviewed studies, this is the generalized percentage attraction range of the compound. This range involved a 66% predicted mean choice percentage, lower than the average value reported for the active compound alone. The maximum proportion value from the meta-analysis indicates an attraction ratio of 2.33*x* for the commercial product, meaning that the active compound combined with the trap will slightly capture more than double the number of individuals. Again, the forest plot shows a similarity of the trial weights but a very high heterogeneity value (I^2^), indicating high variability between the studies (Figure 5). The chi test in the forest plot shows that the overall effect estimated at 66% for this product (diamond in Figure 5) was statistically significant, which indicates that the proportion of individuals who chose the treatment with the compound was significantly different from the control.

**Figure 6:**
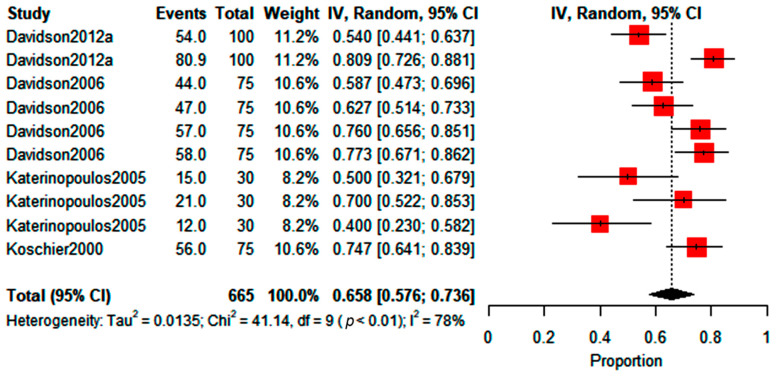
Forest plot for *p-*anisaldehyde showing WFT choosing proportion (random effects model) between treatment with the compound and control. Red boxes represent the point estimate.

In the case of *p-*anisaldehyde (PA), it was recently found that the mixture of this compound with ethyl nicotinate can improve thrips capture in greenhouses, doubling the number of individuals trapped compared to traps without attractants [71]. The forest plot shows that the predicted WFT choosing proportion was found to be between 0.576 and 0.736, being 65.8% of the average percentage attraction of the compound according to the reviewed studies (Figure 6). This average is very similar to the one obtained for Lurem-TR, with an attraction ratio of 2.8x, so this compound could almost triple the number of individuals attracted or trapped. In this case, the heterogeneity value (I^2^) was not as high as that of the other two meta-analyses, but the estimated proportion was statistically significant. Additionally, the funnel plot of the standard error (Supplementary Materials, Figures S1–S3) for all of the analyses (Figure 4, Figure 5 and Figure 6) showed symmetry between the reviewed studies, so there is no evidence of publication bias for the studies included in the meta-analysis.

Finally, a test of the moderator’s regression analysis was carried out with the compound MIN, using as modifiers (1) the type of test (i.e., olfactometer or sticky trap), (2) the study place (i.e., laboratory or field), and (3) the individual type of (i.e., females wild, males wild, and mixed wild). These selected modifiers did not influence the meta-analysis outcome (test type: QM = 1.42, *p-*value = 0.4922; study place: QM = 1.18, *p-*value = 0.2765; and individual type: QM = 3.63, *p-*value = 0.6038), which indicated that the choice proportion toward the treatment with the compound MIN was not significantly affected by such factors.

## 4. Discussion

### 4.1. Trends in the Literature on WFT

Over the past decade, the studies related to WFT increased. These trends showed a growing interest in the scientific community, which lies in the agricultural importance and invasive potential of WFT [72], although its management has focused on searching for and employing synthetic pesticides to regulate thrips populations in affected crops [73,74,75,76,77,78,79]. Unfortunately, the indiscriminate use of these products led to the loss of their effectiveness, due to the resistance processes that generated particular isolated populations and, subsequently, extended to the local/regional levels [75,80,81].

This condition, linked to environmental and worker health issues, has led many studies to focus on generating alternative or complementary tools for controlling thrips. Among the current possibilities, two can be considered the most promising approaches: (1) biological control using beneficial organisms and (2) ethological control using chromatic traps impregnated with semiochemicals. In this context, using the PRISMA approach, we established that over the last 20 years, researchers from various countries joined efforts to find volatile organic compounds and pheromones that serve as attractants for thrips [24], including WFT and other thrips of agricultural importance worldwide, such as the *Thrips tabaci* L. and *T. palmi* Karny (Thysanoptera: Thripidae). Hence, two broad timeframes were recognized for the research on WFT-related semiochemicals. These two periods involved a first period (2000 to 2009) oriented toward collecting information on semiochemical candidates (not precisely from natural origin) that could eventually be used for ethological thrip control, regardless of their origin [24,28,35,40,41,42], and a second period (2010 to 2022) focused on implementing some of the compounds identified in the first period. In the first period, the attempts to recognize the compounds that emit the most attractive host plants to the thrips were unsuccessful, due to a background-collecting phase, as specific compound groups were screened to establish the most attractive hosts to be assessed in ethological control trials [28,35,82,83,84]. In this regard, the transition between the two periods was found to be characterized by the registration and obtention of a pyridine-group-based patent, which was recognized during this investigation stage as the most promising [85].

Consequently, the second period was mainly focused on the effectiveness of MIN in commercial field conditions and different crops [23,25,26,27,31,32,43,45,47,48,67,68,86,87,88,89,90]. Regarding new naturally occurring attractant compounds, some promising candidates were identified, such as (*S*)-(−)-verbenone, 3-hexen-1-ol, 2-phenylethyl acetate, nonanal, (±)-theaspirane and β-caryophyllene [43,91,92,93], and oviposition repellents, such as methyl salicylate, thymol, and carvacrol compounds [89,94,95,96]. Additionally, research integrating biological and ethological control, and even integration with chemical control [97].was found in this period [22,66,90,98]. 

### 4.2. Identified Compounds and Their Attraction Ratio

To date, no systematic review has been published evaluating the attraction ratio of thrips-oriented attractants using a meta-analysis component. This study demonstrates that it is possible to use the knowledge acquired by researchers worldwide to better understand these semiochemical-based alternatives to chemical control and to generate new findings from the information already published. In this sense, it was possible to determine an accurate thrips proportion attracted to the most used compound to date for the ethological control of thrips using meta-analysis as a valuable tool for combined data examination.

Our study found that some compounds, such as *δ*-decalactone, geraniol, *γ*-decalactone, and *o*-anisaldehyde, could have relevant attraction ratios and promising potential for the design of control strategies. However, additional research, mainly under field conditions, is required to confirm this. Lactones have been previously reported as part of the odors of peach fruit, a typical WFT host. These compounds show an attractant effect on the fruit-piercing moth *Eudocima phalonia* [99] and deterrent activity on the peach aphid *Myzus persicae* [100]. Geraniol has a widely known insecticidal and repellent effect on several important insect pests [101], but it is also a flower-derived thrips attractant [102]. Finally, *o*-anisaldehyde belongs to the naturally occurring oxygenated aromatic compounds, which have been reported to exhibit high attractiveness ratio values [28,82]. In parallel to the attractant-focused investigations for WFT, critical studies have been conducted on the pheromones that were previously specified. However, our results show that the attraction rates to pheromone compounds are significantly lower than those obtained for the other evaluated compounds.

Most studies are based on lure-based attractants, which means they are not specific compounds of primary or secondary host plants of the WFT, but were identified from other non-host plants or cross-species attractants [97]. This is consistent, since WFT has been shown not only to respond to volatiles produced by the plant species that primarily attack but also to volatiles from secondary hosts or compounds found in floral fragrances. This is the case for the 4-pyridyl carbonyl group [35,40]. Therefore, most studies avoided the term kairomone and focused more on the attractant concept, so there are attractants from the WFT host plants [103] and attractants derived from other non-host plants.

The attraction ratio values found in this meta-analysis differ from those reported in some studies [35,84] or reported by commercial houses that marketed the compounds, which found values lower than the values estimated in the present study. This can be seen in the MIN’s attraction ratio, which was lower than those for the commercialized product *Lurem-TR*. The meta-analysis collected all variations in the attraction ratio among all of the studies, affording a more conservative value for the compound attraction potential. On the other hand, when each study was analyzed in detail, very high attraction ratio values could be found, and they were not necessarily repeated each time the compound was used again, due to the presence of intrinsic and extrinsic factors related to the system.

Regarding the use of semiochemicals for the control of WFT, in the literature reviewed, in addition to the use of traps, other alternatives were studied, such as the use of secondary host plants established outside or between the crops of interest, even applying other management measures, such as biological or chemical control [70]. For instance, ethological control is compatible with biological control with natural enemies and chemical control using the lure-and-kill approach [104]. Compounds such as methyl jasmonate and *cis*-jasmonate have been recognized [105] as repellants, reducing more than 50% of the feeding of WFT larvae. This constitutes a deterrent effect, causing the insects to leave the plant for other places (i.e., soil), where they can be controlled with other management strategies, including strategies that involve a chemical or biological basis [87].

A word of caution is required regarding the attractiveness values estimated in the present study, which do not directly reflect the compounds’ effectiveness when they are used as part of a management strategy. Instead, they provide an estimate of the attraction level to be expected from their use, either in the form of a compound or a commercial product. Many factors can influence the performance of compounds in the field, generating differences in the attraction values found between the studies. Some of these factors have already been recognized as highly relevant for future research and include extrinsic variables (crops, season, and geographical location) and intrinsic variables (age and feeding) of the investigated species [33,106]. It is also important to note that under open-field conditions, attractant compounds do not appear alone but as part of an odorscape in which background odors can modify the insects’ response [107]. Phenotypic plasticity, through mechanisms such as habituation, could also significantly affect the response to attractants in the field; this is a relevant topic for future research in the case of WFT [108].

A recent topic is the study of WFT-related semiochemicals and their possible applications for controlling this pest. Only 20-year records of those studies are found in the databases, showing the great future potential of such studies. Despite being a topic of recent research interest, the methodologies for the scientific development of these investigations are quite oriented toward a particular compound class–related focus. Thus, it is possible to show that most semiochemical-based studies of the WFT complex have focused on a single compound family, with pyridine-related compounds as the most-studied compound type.

This over-addressing calls attention to the need to strengthen semiochemical-based research in two respects. The first focuses on searching for novel compounds that are capable of generating more attraction than those already studied. This focus will open the possibilities framework for ethological WFT control, maintaining the scientific rigor in the tests that have characterized this study field. However, identifying new compounds exhibiting high attraction ratios is only one step in implementing this alternative as a management strategy [24,25,109]. Future research should also visualize that it is necessary to conduct further studies on the release rate of these active compounds, the types of dispensers used, the radius of attraction, and other formulation factors, depending on the physicochemical characteristics of the compound of interest. The second focus deserving of important research efforts is related to evaluating the biological efficiency and the economic profitability of new semiochemical-based products before commercializing them.

## 5. Conclusions

In addition to pyridine-related compounds, other volatiles with high potential for use in integrated pest management programs have been identified but poorly studied, such as geraniol, *o*-anisaldehyde, *γ*-decalactone, and *δ*-decalactone. These compounds could be used either for the monitoring or mass trapping of WFT. However, few of them have been thoroughly tested in the field or elsewhere, so there is a potential for new research to test and develop products based on these compounds. The most widely used compounds worldwide are those based on pyridines, and they showed a high ratio of attraction of individuals based on integrating field data and laboratory data. Although the Lurem-TR product is based on the compound MIN, they differ in terms of the predicted mean choice percentage values (MIN 76.6% and Lurem-TR 66.6%). The PRISMA approach served to find patterns in the current state-of-the-art research for WFT-related semiochemicals and the research perspectives and directions in upcoming years.

## Figures and Tables

**Table 1:** VOCs reported in the literature as possible WFT attractants.

Compound	Type ^a^	Year RA ^b^	AR ^c^	P(IC) ^d^	T ^e^	Ref.
(−)-(*E*)-caryophyllene	ST	2000	1.03	0.56 * (0.44–0.67)	1	[42]
(−)-*α*-bisabolol	ST	2000	0.97	0.49 (0.38–0.61)	1	[42]
(+) citronellal	MT	2000	0.97	0.49 (0.38–0.61)	1	[42]
(+) citronellol	MT	2000	1.88	0.65 * (0.53–0.76)	1	[42]
(*E*)-*β*-farnesene	ST	2000	1.78	0.64 * (0.52–0.75)	1	[42]
(*E*)-cinnamaldehyde	PP	2000	1.58	0.61 * (0.49–0.72)	1	[42]
(*S*)-(−)-verbenone	MT	2015	2.23	0.68 * (0.63–0.73)	2	[43]
2-isobutyl-3-methoxypyrazine	Ar	2014	0.95	0.48 (0.32–0.65)	2	[26]
3-phenylpropionaldehyde	Ar	2000	1.56	0.56 * (0.44–0.67)	1	[42]
6-amyl-*α*-pyrone	L	2014	1.30	0.54 (0.40–0.67)	3	[26]
benzaldehyde	Ar, AL	2000	2.00	0.67 * (0.55–0.77)	1	[42]
*cis*-jasmone	TD	2014	0.55	0.47 (0.15–0.81)	2	[26]
ethyl-2-chloropyridine-4-carboxylate	Py	2007	1.00	0.58 * (0.44–0.70)	2	[28]
ethyl isonicotinate	Py	2007, 2008	5.98	0.66 * (0.47–0.82)	3	[28,35]
ethyl nicotinate	Py	2000, 2007, 2014	1.81	0.65 * (0.58–0.71)	8	[26,28,42]
eucalyptol	MT	2000	1.14	0.59 * (0.51–0.66)	1	[42]
eugenol	AB	2000	1.88	0.56 * (0.44–0.67)	1	[42]
geraniol	MT	1999, 2000	5.08	0.83 * (0.75–0.88)	3	[41,42]
limonene	MT	2000	1.50	0.60 * (0.48–0.71)	1	[42]
linalool	MT	2000	3.17	0.58 * (0.50–0.66)	1	[42]
Lurem-TR^® f^	Py	2012-2016	2.57	0.75 * (0.74–0.75)	9	[23,31,32,43,44,45,46,47]
Lurem-TR^®^ + Thripline^® f^	Py, Ph ^g^	2016	1.61	0.62 * (0.59–0.65)	1	[46]
*m*-anisaldehyde	Ar	2000	1.34	0.57 * (0.45–0.69)	1	[42]
methyl 4-pyridylketone	Py	2007	3.13	0.67 * (0.56–0.76)	4	[28]
methyl anthranilate	Ar	2014	3.55	0.70 * (0.36–0.91)	2	[26]
methyl isonicotinate (MIN)	Py	2007–2008, 2012–2015	4.95	0.76 * (0.74–0.78)	14	[26,28,29,35,48]
methyl jasmonate	TD	2014	1.55	0.52 (0.20–0.82)	2	[26]
methyl pyrazinoate	Py	2014	1.70	0.68 * (0.55–0.79)	2	[26]
myrcene	MT	1999, 2000	1.16	0.54 * (0.46–0.62)	2	[41,42]
nerol	MT	2000	1.88	0.65 * (0.53–0.76)	1	[42]
*o*-anisaldehyde	Ar	2000	4.00	0.80 * (0.69–0.88	1	[42]
*p*-anisaldehyde	Ar	2000, 2006, 2008, 2012	2.62	0.67 * (0.63–0.70)	6	[35,42,48,49,50]
sabinene	MT	2000	0.63	0.39 (0.28–0.51)	1	[42]
*trans-β*-ocymene	MT	2000	0.85	0.47 (0.35–0.58)	1	[42]
*γ*-decalactone	L	2014	5.17	0.71 * (0.53–0.84)	3	[26]
*γ*-heptalactone	L	2014	1.70	0.63 * (0.39–0.83)	1	[26]
*γ*-nonalactone	L	2014	1.40	0.58 * (0.41–0.73)	1	[26]
*γ*-octalactone	L	2014	1.50	0.60 * (0.44–0.74)	1	[26]
*δ*-decalactone	L	2014	6.57	0.61 * (0.47–0.74)	3	[26]

^a^ Product type of the reported compounds; ST = sesquiterpene, MT = monoterpenoid, PP = phenylpropanoid, TD = terpene derivative, Ar = aromatic, L = lactone, AL = aldehyde, Py = pyridine, AB = allylbenzene, Ph = pheromone. ^b^ Year reported as attractant (RA). ^c^ Attraction ratio (*AR*). For entries with more than one replicate, the mean attraction ratio was calculated. ^d^ Proportion (*P*) of individuals choosing the test compound, and 95% confidence interval (CI); * attraction proportion for the compound is significantly higher (*p* < 0.05) than the obtained for the control treatment according to *x*^2^ test; ^e^ T = trials: Number of bioassays carried out among whole consulted papers. ^f^ Commercial products containing methyl isonicotinate (Lurem-TR^®^) and mixture of methyl isonicotinate and the western flower thrips (WFT) pheromone (Thripline^®^), respectively, including excipients used in the formulation. ^g^ WFT pheromone include two substances, i.e., neryl (*S*)-2-methylbutanoate and *(R)-*lavandulyl acetate (1:0.8 to 1:5 ratios).

**Table 2:** WFT pheromones and attractants. The attraction ratio was calculated between the studies and reported experiments.

Attractants	Type ^a^	Year RA ^b^	*AR* ^c^	*P*(IC) ^d^	T ^e^	Ref.
(*R*)-lavandulyl acetate ((*R*)LA)	E	2005	0.89	0.47 (0.38–0.56)	4	[55]
neryl (*S*)-2-methyl butanoate (N(*S*)2MB)	E	2005	1.49	0.60 * (0.59–0.61)	4	[55]
N(*S*)2MB + (*R*)LA	E, E	2005, 2016	1.58	0.61 * (0.49–0.72)	4	[46,55]
Thripline^® f^	CP	2012-2016	2.49	0.64 * (0.63–0.64)	7	[23,31,46,67,68]

^a^ Product type of the reported attraction sources; A = acetate, E = ester, CP = commercial product. ^b^ Year reported as an attractant (RA). ^c^ Attraction ratio (*AR*). For entries with more than one replicate, the mean attraction ratio was calculated. ^d^ Proportion (*P*) of individuals choosing the test compound, and 95% confidence interval (CI); * attraction proportion for the compound is significantly higher (*p* < 0.05) than the obtained for the control treatment according to *x*^2^ test, ^e^ T = trials: Number of bioassays carried out among whole consulted papers ^f^ Thripline^®^ is a commercial product containing methyl isonicotinate and the thrips pheromone with the excipients used in the formulation. WFT pheromone include two substances, i.e., neryl (*S*)-2-methylbutanoate and *(R)-*lavandulyl acetate (1:0.8 to 1:5 ratios).

## Data Availability

Data may be obtained from the corresponding author upon reasonable request.

## Supplementary Materials

The following supporting information can be downloaded at: https://www.mdpi.com/article/10.3390/insects14030269/s1, PRISMA 2020 checklist, Figure S1: Funnel plot of standard error for methyl isonicotinate meta-analysis, Figure S2: Funnel plot of standard error for *Lurem*-*TR* meta-analysis, Figure S3: Funnel plot of standard error for *p*-anisaldehyde meta-analysis. Ref. [110] are cited in Supplementary Materials (PRISMA 2020 checklist).
