# Supplementary material for: Semiochemicals Associated with the Western Flower Thrips Attraction: A Systematic Literature Review and Meta-Analysis
Source: Insects. 2023 Mar 8;14(3):269. doi: 10.3390/insects14030269 (PMC10053797; doi:10.3390/insects14030269)
Supplement: Supplementary file 1 [file insects-14-00269-s001.zip › Supplementarymaterial.pdf]

Supplementary material

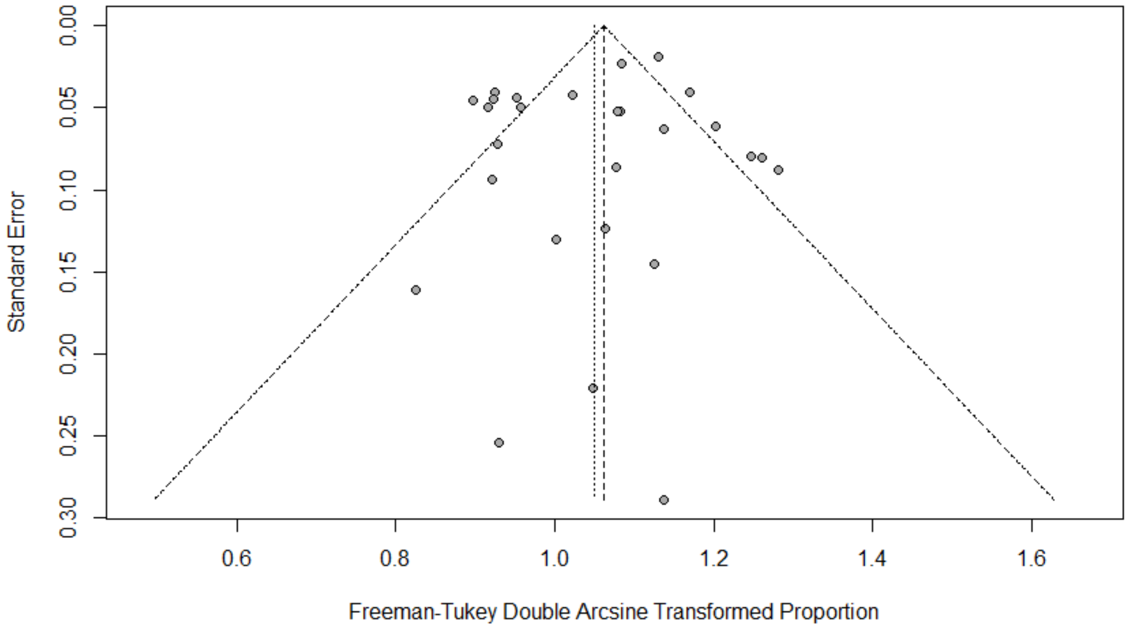

Figure S1. Funnel plot of standard error for methyl isonicotinate meta-analysis.

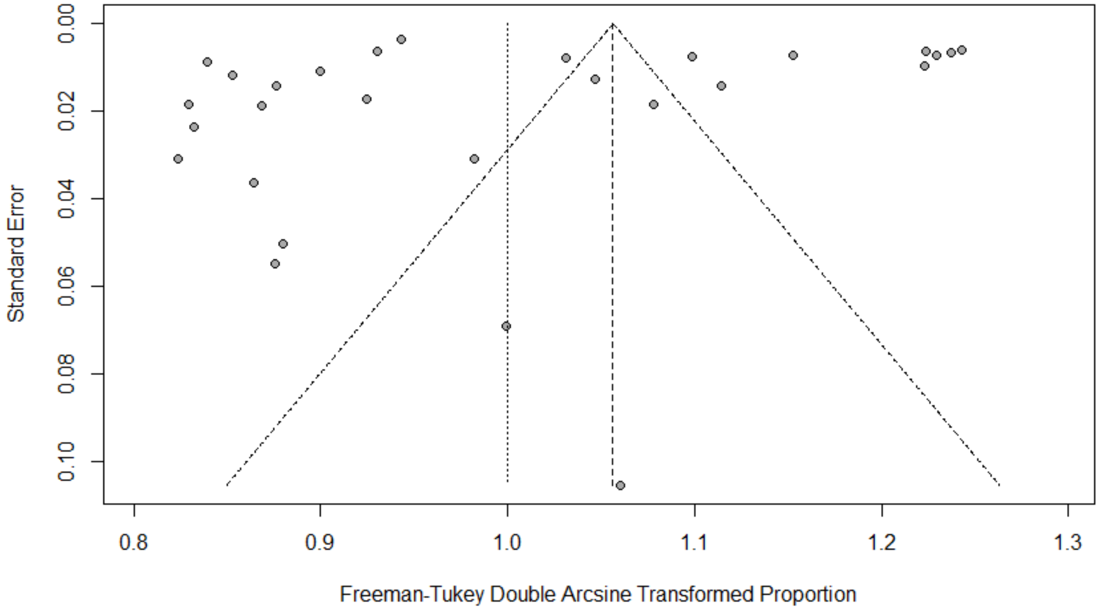

Figure S2. Funnel plot of standard error for Lurem-TR meta-analysis.

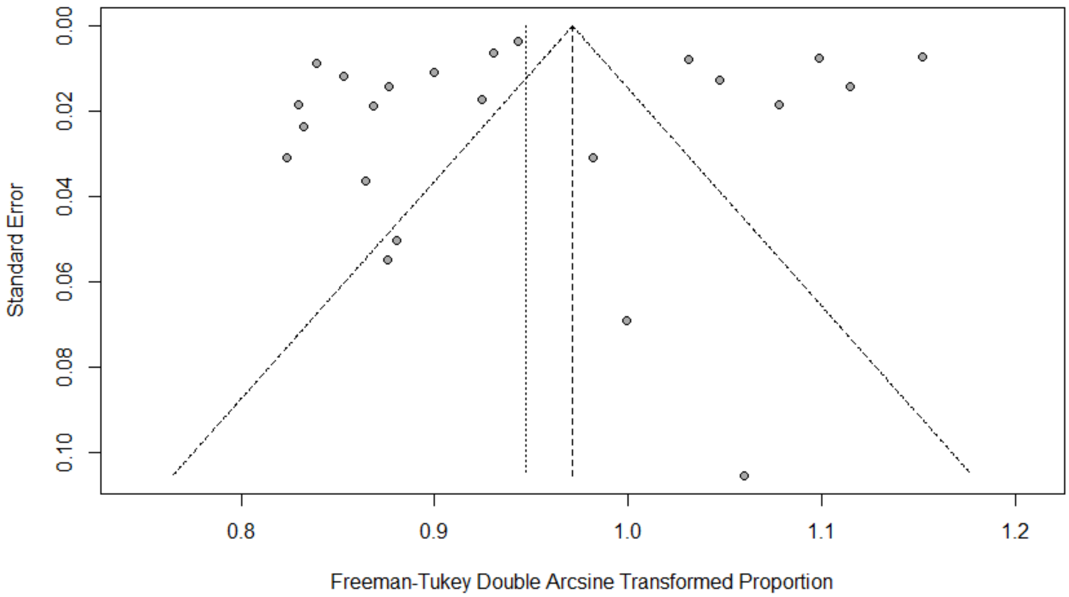

Figure S3. Funnel plot of standard error for *p*-anisaldehyde meta-analysis.
